# Implementation facilitation to promote emergency department-initiated buprenorphine for opioid use disorder: protocol for a hybrid type III effectiveness-implementation study (Project ED HEALTH)

**DOI:** 10.1186/s13012-019-0891-5

**Published:** 2019-05-07

**Authors:** Gail D’Onofrio, E. Jennifer Edelman, Kathryn F. Hawk, Michael V. Pantalon, Marek C. Chawarski, Patricia H. Owens, Shara H. Martel, Paul VanVeldhuisen, Neal Oden, Sean M. Murphy, Kristen Huntley, Patrick G. O’Connor, David A. Fiellin

**Affiliations:** 1 Departments of Emergency Medicine, Yale School of Medicine, New Haven, CT USA; 2 Departments of Internal Medicine, Yale School of Medicine, New Haven, CT USA; 3 Departments of Psychiatry, Yale School of Medicine, New Haven, CT USA; 4 The Emmes Corporation, Rockville, MD USA; 5 Weill Cornell Medical College, New York, NY USA; 6 The National Institute on Drug Abuse, Rockville, MD USA; 7 Yale School of Public Health, New Haven, CT USA

**Keywords:** Buprenorphine, Emergency service, hospital, Opioid-related disorders, Implementation science, Hybrid design

## Abstract

**Background:**

Patients with opioid use disorder (OUD) frequently present to the emergency department (ED) after overdose, or seeking treatment for general medical conditions, their addiction, withdrawal symptoms, or complications of injection drug use, such as soft tissue infections. ED-initiated buprenorphine has been shown to be effective in increasing patient engagement in treatment compared with brief intervention with a facilitated referral or referral alone. However, adoption into practice has lagged behind need. To address this implementation challenge, we are evaluating the impact of implementation facilitation (IF) on the adoption of ED-initiated buprenorphine for OUD into practice.

**Methods:**

This protocol describes a study that is being conducted through the National Institute on Drug Abuse’s Center for the Clinical Trials Network. A hybrid type III effectiveness-implementation study design is used to evaluate the effectiveness of a standard educational dissemination strategy versus IF on implementation (primary) and effectiveness (secondary) outcomes in four urban, academic EDs. Sites start with a standard 60-min “Grand Rounds” educational intervention describing the prevalence of ED patients with OUD, the evidence for opioid agonist treatment and for innovative interventions with ED-initiated buprenorphine; followed by a 1-year baseline evaluation period. Using a modified stepped wedge design, sites are randomly assigned to the IF intervention which is guided by the Promoting Action on Research Implementation in Health Services (PARiHS) framework to assess evidence, context, and facilitation-related factors impacting the adoption of ED-initiated buprenorphine. During the 6 months of IF through the 1-year IF evaluation period, external facilitators work with local stakeholders to tailor and refine a bundle of activities to meet the site’s needs. The primary analyses compare the baseline evaluation period to the IF evaluation period (*n* = 120 patients with untreated OUD enrolled during each period) on (1) rates of provision of ED-initiated buprenorphine by ED providers with referral for ongoing medication (implementation outcome) and (2) rates of patient engagement in addiction treatment on the 30th day after the ED visit (effectiveness outcome). Finally, we will perform a cost-effectiveness analysis (CEA) to determine if the effectiveness benefits are worth the additional costs.

**Discussion:**

Results will generate novel information regarding the impact of IF as a strategy to promote ED-initiated buprenorphine.

**Trial registration:**

ClinicalTrials.gov NCT03023930 first posted 1/10/2017, https://clinicaltrials.gov/ct2/show/NCT03023930?term=0069&rank=1

**Electronic supplementary material:**

The online version of this article (10.1186/s13012-019-0891-5) contains supplementary material, which is available to authorized users.

## Background

Untreated opioid use disorder (OUD) is a public health problem in the USA despite the availability of medications for OUD (MOUD) [1] [2], with demonstrated effectiveness. Patients with untreated OUD frequently receive care in emergency departments (EDs) for treatment of acute and comorbid medical conditions, opioid overdose, withdrawal, and seeking access to addiction treatment. ED visits for opioid overdose increased 30% from 2016 to 2107 [3]. EDs typically provide community referrals, including to opioid treatment programs (OTPs), for OUD treatment rather than initiating treatment with medications such as buprenorphine. We demonstrated that among patients with untreated OUD seen in the ED for varied reasons, ED-initiated buprenorphine with referral to ongoing buprenorphine and medication management was superior to strategies that did not initiate buprenorphine treatment. Patients who were initiated on buprenorphine in the ED were nearly twice as likely to be engaged in treatment on the 30th day post ED visit and reported less illicit opioid use [4], and ED-initiated buprenorphine was cost effective [5].

Despite this evidence, there is a profound implementation gap [6]. An analysis of 17,568 individuals with non-fatal opioid overdoses demonstrated that only one in three patients with OUD subsequently received the most effective treatment, MOUD. Nearly 5% of non-fatal overdose survivors died within 1 year, and all-cause and opioid-related mortality was significantly reduced if the survivors received treatment with buprenorphine or methadone [7], thus highlighting the urgency and necessity of implementing ED-initiated buprenorphine.

Research is needed to determine the most effective strategies to implement ED-initiated buprenorphine with referral for ongoing MOUD in real-world ED settings. Standard implementation strategies rely on educational formats such as Grand Rounds presentations. Implementation facilitation (IF) is a robust strategy with success at promoting evidence-based practices in diverse clinical settings [8, 9]. It is described as “helping individuals and teams to understand what they need to change and how they need to change to apply evidence to practice [8, 10].” External facilitators working with local stakeholders tailor and refine a bundle of activities to meet the site’s needs [11, 12].

This manuscript describes the protocol of a study designed to determine the impact of a standard educational strategy versus IF on the provision of ED-initiated buprenorphine with referral for ongoing MOUD. To simultaneously assess effectiveness, we used a hybrid type III effectiveness-implementation design [10] and assessed the rates of patient engagement in formal addiction treatment on the 30th day post ED visit.

## Methods/design

### Overview (see Fig. 1)

This multisite study, funded by the National Institute on Drug Abuse’s Center for the Clinical Trials Network (NIDA CTN), uses a modified stepped wedge design [10] to randomize four geographically diverse ED sites to calendar time during which they would undergo a sequence of evaluation and implementation (Fig. 2). The first is a standard educational dissemination activity using a “Grand Rounds” format followed by 12 months of a baseline evaluation period. During this time, 120 patients with moderate to severe OUD are enrolled at each site. The second is a 6-month intensive IF period. The IF period is followed by a 12-month IF evaluation period during which a second group of 120 patients with moderate to severe OUD are enrolled at each site. The primary analysis compares the baseline evaluation period to the IF evaluation period on [1] implementation—the rates of provision of ED-initiated buprenorphine with referral for ongoing MOUD and [2] effectiveness—the proportion of patients engaged in formal OUD treatment on the 30th day post ED. As the efficacy and effectiveness of buprenorphine and addiction treatment are well established, we use a hybrid type III effectiveness-implementation design and identified the implementation outcome as primary [10].

**Fig. 1:**
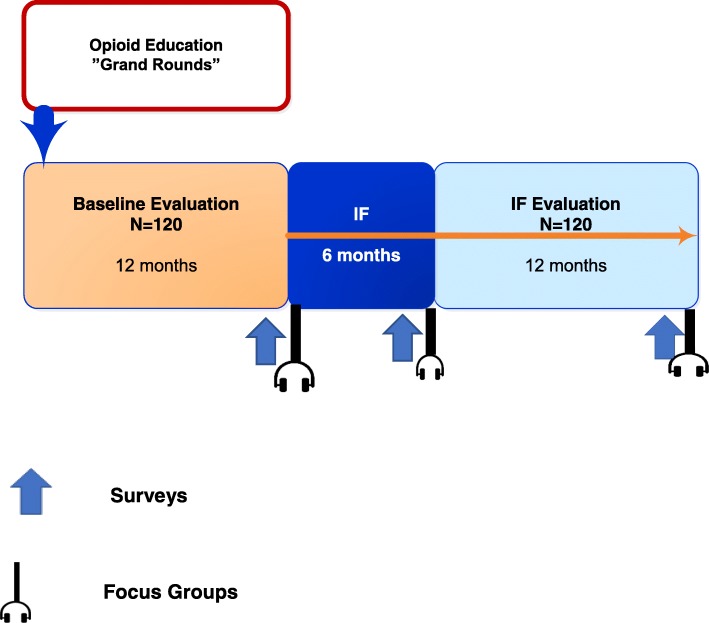
Study activities overview

**Fig. 2:**
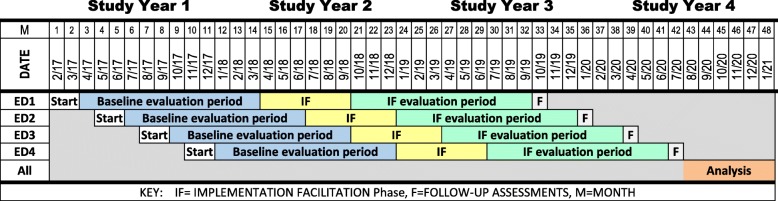
Timeline

### Site selection

Site selection occurred through a formal application and review process. NIDA CTN Nodes and affiliates received a synopsis of the protocol and completed a standard form. Selection was based on [1] the prevalence of patients with International Classification of Diseases 9 and 10 codes reflecting opioid-related diagnoses, ensuring enrollment targets of 10–12 patients per month; [2] ED investigators with experience conducting federally funded research; [3] ED characteristics, including the presence of an electronic health record (EHR) that can be queried routinely, and buprenorphine on the hospital/ED formulary; [4] a sufficient number of community OUD treatment providers/programs that could accommodate patients referred from the ED within 96 h [5] access to pharmacies within proximity of the ED to fill buprenorphine prescriptions and [6] lack of existing ED-initiated buprenorphine activities. All sites agreed to a central institutional review board. Four geographically diverse academic, urban ED sites were chosen from 10 applicants: Johns Hopkins Hospital in Baltimore, MD; Mount Sinai (two locations—The Mount Sinai Hospital and Mount Sinai Beth Israel Hospital) in New York City, NY; University of Cincinnati Medical Center in Cincinnati, OH; and Harborview Medical Center in Seattle, WA.

### Study participants

The study includes two distinct participant groups who participate in both quantitative and quantitative research—[1] ED and community-based providers and stakeholders, and [2] patients with OUD.

#### Provider participants

ED providers include attending physicians and residents, advanced practice providers (physician assistants, advanced nurse practitioners), nurses, counselors, social workers, pharmacists, and administrators. The community providers consist of physicians, advanced practice providers, administrative leaders, counselors, and social workers who work at a range of programs providing treatment for OUD. The study requires that there be at least one federally licensed OTP (e.g., methadone clinic) and office-based practice that prescribed buprenorphine at each site.

#### Patient participants

Patients are screened and enrolled on ED shifts during baseline and IF evaluation periods. During the IF period, patients receive standard ED care, and no enrollment occurs (Fig. 1). Patients are eligible for enrollment if > 18 years of age, meet Diagnostic and Statistical Manual for Mental Disorders, Fifth Edition (DSM-5) criteria for moderate/severe OUD, have a positive urine test for opioids (including methadone or buprenorphine), understand English, and consent to study procedures. Since testing for fentanyl is not routinely available in EDs, and there is no FDA-approved fentanyl point of care testing for clinical care, individuals testing positive for fentanyl on “forensic use only” toxicology strips are not eligible. Patients are excluded if they have a medical or psychiatric condition requiring hospitalization at the index ED visit, are actively suicidal, are cognitively impaired, present from an extended care facility, require opioids for a pain condition, or are currently (past 30 days) enrolled in a formal OUD treatment program (excluding mutual help or 12-step programs). Patients can only be enrolled into the study once, irrespective of the evaluation period. Patients completing their 30-day outcome assessment are eligible to participate in the focus groups in the IF formative evaluation (see below). Patient participants receive a $50 gift card for completing the enrollment process at the initial ED visit and $50 card for the 30-day post enrollment evaluation. Study patients who participate in the focus groups are provided a meal and receive a $50 gift card for their time.

### Study assessments

The Promoting Action on Research Implementation (PARiHS) framework guides our assessments [13].

#### Site assessments

Each site completes assessments describing their ED, hospital, and community treatment programs. This includes each organization’s size, location, payer mix, existing substance use services, and follow-up resources; the range and number of treatment services in the ED catchment area; and ED provider characteristics including full-time equivalents, age, sex, years, and level of training.

### Provider assessments

#### Quantitative data: ED and community provider stakeholder surveys

Anonymous web-based surveys are collected via email at three distinct timepoints; (1) last 6 weeks of the baseline evaluation, (2) first 6 weeks of the IF evaluation period, and (3) last 6 weeks of the IF evaluation period. Each stakeholder receives a customized introductory email from their respective leadership explaining the purpose of the survey, the time allotment for completion, and the title of the email they will receive with the link to the survey. The survey completion is considered evidence of consent for the respondent’s information to be reported in aggregate. Reminders are sent to non-responders weekly, and the survey is open for 6 weeks. To avoid IF activities impacting survey responses, survey collection is completed prior to the initiation of IF. We use an adapted Organizational Readiness to Change Assessment (ORCA) modified to focus on ED-initiation of buprenorphine (ED ORCA) [14] and adapted provider readiness and preparedness to change “rulers,” based on previous work suggesting the importance of assessing physician stage of change [15] with a single item, without sacrificing reliability and validity [16]. The ORCA has been applied to the evaluation of interventions intended to promote evidence-based practices, including addiction treatment, and predicts implementation efforts [17, 18]. Baseline ORCA, and provider readiness and preparedness scores are used by external facilitators as part of the formative evaluation (see description of IF, Table 1) to determine evidence and context-related strengths and weaknesses in organizational readiness to implement buprenorphine and referral to treatment and to tailor the IF.

**Table 1:** Implementation facilitation activities

Activity	Definition
External facilitators	The investigators in fields of emergency medicine, internal medicine, addiction medicine, and psychology provide expertise in buprenorphine, ED interventions, referral to community services, and techniques related to brief interventions and motivate patients to engage in treatment. External facilitators (EF) lead the focus groups and meet with the site leaders and local champions through individual and group face-to-face meetings at the beginning of IF, and again at 4–5 month into the IF period. Additional calls and emails occur throughout the IF and IF evaluation periods. The EF team meets weekly for 3–4 h and reviews site enrollment, survey completions, and IF activities. All IF activities are cataloged into a data repository. Site checklists (Additional file 1) are reviewed monthly and plans are made to facilitate any needed activities.
Formative evaluation	Anonymous surveys of ED and community providers, followed by focus groups with key ED, community provider, and patient stakeholders in conjunction with one-on-one individual meetings with designated administrative leaders, perceived role models, and opinion leaders provide insights into individuals and organizational readiness, power for change, and ideas to move adoption forward.
Local champions	Local site champions self-identify or are identified by ED or community providers in the focus groups and are recruited to participate. One-on-one discussions occur, and information requested or considered important for each group is emailed routinely. These individuals support activities essential for adoption of ED-initiated buprenorphine. Local champions are not paid by the study to ensure sustainability of practice adoption and reproducibility of the findings.
Stakeholder engagement	Data from the surveys and focus groups are compiled into the domains of evidence, context, and facilitation and shared with local champions and ED and community administrative leaders. The EF team seeks to align ED-initiated buprenorphine with local priorities. A site IF team then forms consisting ideally of champions and leaders with the ability to support change.
Tailor program to site	The EF works with the IF team to tailor their interventions specific to their site based on information gathered during the formative evaluation and all procedures and processes are incorporated into the site’s ED workflow
Provider education and academic detailing (AD)	Efforts are made to facilitate trainings by leveraging local expertise at the sites. Sites are notified of local DATA 2000 (X waiver) trainings and provided information on how to schedule a training. An academic detailing pamphlet was developed to inform the providers specifically noting: Why the ED; what is the evidence; why use buprenorphine; and “how to” treatment algorithms and referral/discharge forms are included. Journal club articles and websites are provided. The EF team provides AD to champions, thought leaders, and providers throughout the IF and IF evaluation period.
Performance monitoring and feedback	The identified lead local champion periodically completes a checklist provided as reporting items as number of providers with X waivers, status of ED policy and procedures for initiating buprenorphine, any EHR upgrades to enhance adoption, presence of formal agreements with community sites to accept ED referral (see Additional file 1 for the complete list). Sites are encouraged to provide feedback to providers when a patient engages in treatment and perform routine departmental quality performance efforts reporting number of patients with OUD administered or prescribed buprenorphine.
Learning collaborative (LC)	Sites are invited to join a monthly Learning Collaborative at the start of the IF period. Lessons learned from participating sites are discussed addressing facilitators and barriers to local processes. The EF team also invites outside experts and champions to discuss their adoption processes. Email listservs are developed and distributed bringing together multidisciplinary site champions as well as like disciplines among sites, such as advanced practice practitioners and residents.
Problem-solving	The EF routinely discusses issues regarding ED, organizational, and community barriers with site champions. Innovative problem-solving ideas and activities are shared in the LC. The EF provides other consultations and materials as needed.
Program marketing	Efforts designed to increase attention to ED-initiated buprenorphine. Handouts as well as pens and pins denote the messages of the “Project ED Health” such as “Buprenorphine saves lives” and “Buprenorphine treatment works” and sites are referred to websites the EF team developed to retrieve information for patients and providers [19, 20].

The ORCAs for ED and community providers ask the respondent to rate local factors related to evidence, context, and facilitation on a 5-point Likert scale (strongly disagree to strongly agree or very infrequently to very frequently) (Additional file 3). Facilitation questions are omitted from the survey conducted at the end of baseline assessment since the IF has not yet taken place.

The change rulers assessing the providers’ readiness and preparedness to provide ED-initiated buprenorphine with referral to ongoing care are measured on a continuous scale from 0 to 10.

#### Qualitative data: focus groups

We conduct focus groups at each site with a purposeful sample of key ED, community, and patient stakeholders at three timepoints: during the first month of the IF period, during the fourth to fifth months of the IF period, and within the last month of the IF evaluation period. Focus groups are conducted with approximately four to eight representatives from each of the stakeholder categories, including emergency physicians, residents, advanced practice practitioners, nursing, social workers, case managers, ED leadership, pharmacists, community providers, community administrators, and patients with OUD who have received ED care [21]. The narrative for the focus groups changes as the IF period progresses but is based on the PARiHS framework [14]. The scripts that guide the focus group discussions are included in Additional file 1. To inform IF activities in real time, we used the Rapid Assessment Process [22], a type of participatory action research using intensive, team interaction, and multiple cycles of data collection followed by data review and analysis. We estimate that approximately 8–10 events will occur until themes begin to repeat. This process allows for results to be used for planning, monitoring, and evaluating activities when prolonged fieldwork usually associated with traditional qualitative research is not possible.

### Implementation interventions

#### Standard educational intervention

A traditional 60-min Grand Rounds presentation occurs as part of the site’s existing ED educational series at the start of the baseline evaluation period. Attendance is encouraged and advertised like other department grand rounds. The content is consistent across sites, except for local OUD and overdose epidemiology, delivered by the same presenter (GD), and includes 50 min of didactics and 10 min for questions and discussion. The content includes as follows: OUD and overdose epidemiology; evidence supporting the use of opioid agonists, evidence for ED-initiated buprenorphine [4]; how MOUD reduced opioid overdose deaths, safe prescribing of opioids, overdose education, and naloxone distribution; and the stigma of addiction. Specifics of “How to” develop an ED-initiated buprenorphine program are not included in the 50-min presentation, but available and displayed based on request. No information regarding ED-initiated buprenorphine was withheld if requested.

#### Implementation facilitation procedures

The IF is based on the manualized program developed by Kirchner and colleagues [22] (https://www.queri.research.va.gov/tools/implementation/Facilitation-Manual.pdf) that has had significant impact on implementing healthcare practices. The manual (https://www.queri.research.va.gov/tools/implementation/Facilitation-Manual.pdf) was modified for ED-initiated buprenorphine. A central aspect of IF includes the role of the external facilitators working with local stakeholders. The components of the IF used in this study are outlined in detail in Table 1. Of note, local champions are not paid by the study to ensure sustainability of practice adoption and reproducibility of the findings. IF activities are iterative and continue into the IF evaluation period.

The three-stage formative evaluation adapted from the PARiHS framework [13] uses a mixed-methods approach to identify evidence, context, and facilitation-related factors impacting the provision of ED-initiated buprenorphine with referral for ongoing MOUD in the community. Understanding the site-specific practices, as well as facilitators and barriers to changing practice identified by the stakeholders through surveys, focus groups and one-on-one meetings, and phone calls, informs specific interventions that are then tailored to the site. Strategies are refined and enhanced in an iterative manner to improve implementation success.

#### Resource development

The external facilitation team develops resources needed for ED adoption of ED-initiated buprenorphine, including an integration pathway into ED flow, treatment algorithms, patient assessments for OUD and the Clinical Opioid Withdrawal Scale (COWS), discharge instructions for unobserved home induction and referral forms; and provider education including slide sets, references, and an academic detailing brochure. All materials reside on NIDA’s [19] and the external facilitation team’s institution [20] websites. The sites tailor these materials to their individual EDs and community needs.

### Measures (implementation)

Process measurements include ED provider obtainment of Drug Addiction Treatment Act of 2000 (DATA 2000) waivers required to prescribe (but not dispense) buprenorphine, adherence to Critical Action Checklist in prescribing and referring, and organizational data including workflow integration into the EHR. A local champion at each site completes a performance monitoring checklist that catalogs their progress in adopting ED-initiated buprenorphine into their practice (see Additional file 2). The primary implementation outcome, ED-initiated buprenorphine with referral for ongoing MOUD, is abstracted from the medical record using the ED Visit Review Form. The patient’s chief complaint, discharge diagnoses, medications administered and/or prescribed, and discharge instructions regarding direct linkages with community treatment providers and programs are recorded by a trained lead RA. A second reviewer (site PI) reviews the data collection form for the first 10 patients for concordance with the lead RA, followed by review of every fourth enrollment with the goal of 25% of forms completed at their site. Non-concordance triggers a more in-depth lead RA learning process and a second review involving the four site PIs.

### Patient screening, eligibility, and assessments (baseline and IF evaluation periods)

#### Screening and eligibility determination

The screening and follow-up assessments are designed to be brief, balancing the value of comprehensive data against feasibility in the busy ED environment and minimizing assessment reactivity [23]. Patients are screened by research associates (RAs) for eligibility during any phase of their ED visit. There was an attempt to identify patients early in their ED visit to minimize the impact of research on length of stay. Screening is conducted after verbal consent. The screener includes questions about prescription and illicit opioid use in the past 30 days embedded in questions about general health and substance use [24, 25]. Potential study patients who report any opioid use in the past month complete a 7-day timeline followback (TLFB) [26]. If opioid use is reported during the past 7 days, they undergo a structured diagnostic interview (DSM-5) to evaluate for the presence of moderate to severe OUD. Potential study patients who do not meet criteria for moderate to severe OUD are given a handout recommending that they abstain from drug use and a list of local referral options at the discretion of their ED providers. Those who meet criteria for moderate to severe OUD are informed that they may qualify for a study and asked to produce a urine sample. If the urine tests are positive for any opioid (patients whose urine is positive only for fentanyl are not *eligible*), the patient is asked to provide contact information for two separate contacts and is offered participation. Eligible patients are asked to provide written consent for all study procedures and assessments including completing a separate medical record release form to contact a treatment provider at 30 days post ED visit.

#### Patient assessments (see Table 2)

In addition to demographic and clinical characteristics and locator information, the baseline data includes a brief instrument assessing health status, healthcare utilization, overdose, alcohol, and drug use, the EuroQol (EQ-5D) [27]. The total time burden for the baseline assessments is less than 30 min. Follow-up assessments collected at 30 days post index ED visit are similar. Engagement in formal addiction treatment on their 30th day post enrollment is operationalized such that the ED enrollment visit is day 0. Those who report being engaged in treatment report the type of treatment, i.e., methadone, buprenorphine and/or naltrexone treatment, detoxification, residential, or inpatient treatment. Type of treatment is classified according to the American Society of Addiction Medicine (ASAM) Levels of Care [28]. All patient reports of treatment engagement on the 30th day are verified through contact with the treatment facility. Lastly, an ED visit and hospitalization form collects information regarding any ED visits and/or hospitalizations.

**Table 2:** Schedule of patient activities and assessments by time period

Instrument/activity	Time	Done by	Baseline evaluation period (12 months)			IF (6 months)	IF evaluation period (12 months)		
			Screening	Enrollment	30-day follow-up		Screening	Enrollment	30-day follow-up
			(Index ED visit 1)		(Visit 2)		(Index ED visit 1)		(Visit 2)
ED health quiz	2′	RA	X				X		
DSM-5	5′	RA	X				X		
Urine drug screen	5′	RA	X				X		
Patient eligibility summary	2′	RA	X				X		
Written compound informed consent	10’	RA		X				X	
Demographics and additional characteristics	1’	RA		X				X	
Locator information form	2’	RA		X				X	
Other substance use	1’	RA		X				X	
Timeline followback (TLFB)	10’	RA		X	X			X	X
Health services utilization (inpatient and outpatient)	6’	RA		X	X			X	X
Health status (HRBS/PHQ9/PEG)	3’	RA		X	X			X	X
Overdose	1’	RA		X	X			X	X
EuroQol-5D	2’	RA		X	X			X	X
Crime and criminal justice	1’	RA		X	X			X	X
Urine drug screen	5’	RA			X				X
Healthcare visit logistics	1’	RA			X				X
Engagement in treatment	5’	RA			X				X
ED visit review		RA		X				X	
Critical action checklist		RA^a^		X				X	
ED visits and hospitalizations		RA			X				X
Study completion		RA			X				X
Serious adverse event (death)		RA	As needed				As needed		
Protocol deviations		RA	As needed				As needed		
Total duration in minutes for participant interaction:			14’	37’	34’		14’	37’	34’

^a^Site staff, with PI input as needed

## Statistical analyses

### Primary implementation and effectiveness aims and outcomes

Consistent with a hybrid type III effectiveness-implementation design, the primary research question compares the impact of the standard educational intervention to that of the IF on implementation of ED-initiated buprenorphine with referral for ongoing MOUD. We also evaluate the relative impact of the two implementation interventions on engagement in formal addiction treatment. The primary implementation outcome compares the baseline evaluation period and the IF evaluation period on rates (proportions) of provision of ED-initiated buprenorphine with referral for ongoing MOUD. We hypothesize that the rates of provision of ED-initiated buprenorphine with referral for ongoing MOUD will be significantly higher during the IF evaluation period compared with the baseline evaluation period. This outcome was used to determine the study’s sample size.

The effectiveness outcome compares the baseline evaluation period and the IF evaluation periods on rates of patient engagement in formal addiction treatment on the 30th day after the ED visit. We hypothesize that the proportion of patients during the IF evaluation period engaged in formal addiction treatment on the 30th day post ED visit will be significantly higher than during the baseline evaluation period. Engagement in formal addiction treatment will be assessed by direct contact with the facility and/or treating clinician. Formal addiction treatments are those treatments consistent with the ASAM levels of care (1–4) and can include a range of clinical settings including office-based providers of buprenorphine or naltrexone, federally licensed opioid treatment programs (OTP), intensive outpatient, inpatient, or residential treatments. Of note, as patient preference and provider clinical judgment can impact the type of treatment that a patient receives at a given time, we do not require that patients were receiving MOUD on the 30th day following the index ED visit to be considered engaged in formal addiction treatment. However, participation in a self-help program, e.g., Narcotics Anonymous (NA), alone will not be considered as engagement in formal addiction treatment. “Sober home” residence, often unregulated and unaccredited, will not be considered alone as enrolled in formal addiction treatment.

### Secondary and exploratory outcomes

Secondary and exploratory outcomes related to implementation include provider and organizational readiness and preparedness to adopt ED-initiated buprenorphine with referral to treatment, and fidelity to the process of initiating buprenorphine. Secondary patient outcomes such as overdose events, HIV risk-taking behaviors, health status (i.e., other substance use, co-morbid conditions such as hepatitis C and HIV), depression using the Patient Health Questionnaire-9 (PHQ-9) [29] screen, pain intensity, and interference with enjoyment of life and general activity (PEG) [30], healthcare service utilization, and crime and criminal justice involvement are assessed at 30 days and described in detail in Table 3.

**Table 3:** Secondary outcomes and analytic plans

Implementation outcomes	
Fidelity as measured by adherence to a critical action checklist regarding the provision of ED-initiated buprenorphine with referral for ongoing MOUD using standard models including mixed models, or other appropriate methodology.	
Rates of enrolled patients receiving an appointment for opioid treatment provider/program upon ED discharge using the model described for the primary analysis.	
Number of ED providers receiving DATA 2000 training using appropriate methodology. Permutation tests will be considered for this analysis.	
Number of clinicians providing ED-initiated buprenorphine with referral for ongoing MOUD using count models. Models considered will be Poisson, zero-inflated Poisson, negative binomial, and zero-inflated negative binomial. An offset will be considered for how long the provider has been trained.	
ED provider readiness and preparedness ruler scores to initiate buprenorphine and provide referral for ongoing MOUD using standard models including mixed models or other appropriate methodology.	
ED Organizational Readiness to Change Assessment (ORCA) scores relating to ED-initiated buprenorphine with referral for ongoing MOUD using standard models including mixed models or other appropriate methodology.	
Community opioid treatment provider/program readiness and preparedness ruler scores to continue MOUD for patients with OUD who have received ED-initiated buprenorphine using standard models including mixed models or other appropriate methodology.	
Community opioid treatment provider/program Organizational Readiness to Change Assessment (ORCA) scores relating to receiving patients with OUD who have received ED-initiated buprenorphine using standard models including mixed models or other appropriate methodology	
Effectiveness outcomes	
Self-reported days of illicit opioid use (past 7 days) as measured by TLFB methods at 30 days using count models. Models considered will be Poisson, zero-inflated Poisson, negative binomial, and zero-inflated negative binomial.	
Overdose events (past 30 days) captured by participant self-report, state medical examiner records, National Death Index, and review of medical records will be compared using count models. Models considered will be Poisson, zero-inflated Poisson, negative binomial, and zero-inflated negative binomial. An offset will be considered for how long a participant was in a given period if less than 30 days.	
HIV risk-taking behaviors (past 30 days) as measured by HIV Risk Taking Behavior Score using standard models such as mixed models or other appropriate methodology.	
Healthcare service utilization (past 30 days) measured by Health Services Utilization Form will be compared using count models. Models considered will be Poisson, zero-inflated Poisson, negative binomial, and zero-inflated negative binomial. An offset will be considered for how long a participant was in a given period if less than 30 days.	
Rates of illicit opioid negative urines at 30 days using the model described for the primary analysis in section.	

### Statistical considerations

The following model will be used to compare the IF evaluation period to the baseline evaluation period for the rates of ED-initiated buprenorphine with referral for ongoing MOUD (primary study and primary implementation outcome). The same model will be used to compare the rates of patient engagement in formal addiction treatment on the 30th day after ED visit (effectiveness outcome).

logit(*p*_*si*_) = *α* + *βt*_*si*_ + *γf*_*si*_ + *r*_*s*_∗

[*p*_*si*_ is the probability of success of patient *i* at site *s*; *t*_*si*_ is the calendar time of enrollment of patient *i* at site *s*; *f*_*si*_ is the indicator of whether patient *i* at site *s* is in the baseline evaluation period (*f* = 0) or IF evaluation period (*f* = 1); and *r*_*s*_ is the random effect of site *s*, where *r*_*s*_~*N*(0, *σ*)].

In this model, the *γ* estimate represents the estimated difference in the logit of the probability of success. The model will test a one-tailed hypothesis at a 0.05 level. The null and alternative hypotheses are *H*_0_ : *γ* = 0 *H*_*a*_ : *γ* > 0.

Several covariates may affect the effectiveness outcome. At a minimum, sex, race, and ethnicity will be considered as covariates in the model. They will be entered into the model in a stepwise manner using a *p* value cutoff of 0.05. The most complicated model with the three minimal covariates would be:

$$ {\displaystyle \begin{array}{l}\mathrm{logit}\left({p}_{si}\right)=\alpha +{\beta t}_{si}+{\gamma f}_{si}+{{\boldsymbol{\theta}}_1}^{\ast }{\mathrm{sex}}_{si}+{{\boldsymbol{\theta}}_2}^{\ast }{\mathrm{race}}_{si}+{{\boldsymbol{\theta}}_3}^{\ast }{\mathrm{ethnicity}}_{\boldsymbol{si}}\kern0.5em \\ {}+{\theta_4}^{\ast }f\;{si}^{\ast}\;{\mathrm{sex}}_{si}+{\theta_5}^{\ast}\;{\mathrm{race}}_{si}+{\theta_6}^{\ast }f\;{si}^{\ast}\;{\mathrm{ethnicity}}_{si}+{r}_s\end{array}} $$

where sex, race, and ethnicity are indicators for their respective variables. Additional appropriate covariates and parameters may also be considered in the above model.

### Secondary outcomes

Secondary outcomes will be compared in the IF evaluation period to the baseline evaluation period. For all secondary effectiveness outcomes, covariates such as sex, race, and ethnicity will be considered if appropriate and feasible. Specifically, if model-based approaches are used, covariates will be considered. If non-parametric methods are used, then the tests may be done at different covariate levels. If the data do not follow a Gaussian distribution, a modified Wilcoxon test will be used where needed [31].

### Exploratory analyses

We will evaluate a set of provider, site and patient characteristics for their effect on the implementation and effectiveness outcomes. ED characteristics including size, location, existing substance use services and resources, and type and number of addiction treatment services in the ED’s catchment area will be described. ED provider characteristics including age, sex, years, and level of training will be evaluated. These analyses will use similar models as for the primary analysis, the MIXED models procedure repeated measures and generalized estimating equations (GEE), or other appropriate regression, clustering, and factor analytical tools to evaluate potential impact of site factors and patient characteristics on the implementation and effectiveness outcomes. Patient characteristics will include sex, race/ethnicity, health insurance status, age, primary drug (heroin versus prescription opioids), reason for presentation such as seeking treatment for OUD or overdose, referral site, and pain intensity and interference.

#### Cost-effectiveness of standard educational intervention versus implementation facilitation

The cost-effectiveness analysis will be conducted from the perspectives of the healthcare sector and society [32]. The healthcare sector perspective includes all formal healthcare costs incurred on behalf of the participant, while the societal perspective encompasses all costs associated with the intervention, including those in the healthcare sector, regardless of who incurs them. Thus, the healthcare sector perspective includes those associated with the intervention, the cost of buprenorphine, and all downstream medical costs. The startup and day-to-day administration costs of IF will be calculated using a combination of macrocosting and microcosting techniques applied during semi-structured interviews with site personnel. The Drug Abuse Treatment Cost Analysis Program (DATCAP) instrument will guide this process [33, 34]. Startup costs will be reported separately, as they become negligible over time, and research costs will be excluded because they would not be incurred in standard care. The societal perspective will include all costs from the healthcare sector and costs associated with patient participation in treatment (e.g., time, transportation), criminal activity, and reduced workplace or educational productivity. This information will be collected by self-report through a resource utilization survey. In accord with cost-effectiveness analyses guidelines, unit costs will, to the best of our ability, reflect the actual value of the resources [32]. Healthcare services will be valued using nationally representative Medicare reimbursement rates derived from the Medical Expenditure Panel Survey [35]. The Federal Supply Schedule will be used to value pharmaceuticals [32].

We will evaluate three measures of cost-effectiveness: a process measure, a clinical measure, and an economic measure. Our process measure is participant engagement in formal OUD treatment day 30. Time abstinent from opioids, operationalized as proportion of the year abstinent from opioids, will serve as the clinical measure of effectiveness and allows for comparisons with other economic evaluations of OUD interventions [36]. The economic outcome will be the quality-adjusted life year (QALY) [37, 38].

Incremental cost-effectiveness ratios will be calculated, defined as Δ*C*/Δ*E*, where Δ*C* is the difference in mean costs and Δ*E* is the difference in mean effectiveness between the baseline evaluation period and IF evaluation period. We will calculate predicted mean costs using generalized linear mixed models which allow the appropriate mean and variance functions to be chosen according to the observed data and allow for random effects.

Uncertainty around the incremental cost-effectiveness ratios will be assessed using cost-effectiveness acceptability curves, which indicate the probability that the intervention is cost-effective at different willingness to pay threshold values. For cost estimates, subject to debate either because of known imprecision in the estimation procedures or lack of adequate information, we will conduct sensitivity analyses.

We will use appropriate non-parametric, parametric, and analysis of variance statistical procedures to descriptively evaluate the key characteristics of each site, indicators of organizational-level differences between the sites (e.g., the number ED providers, number/ratio ED providers DEA waivered to prescribe buprenorphine), and to evaluate comparability of baseline characteristics among patient cohorts enrolled at each of the sites and overall during baseline evaluation period and the IF evaluation periods across all sites.

## Discussion

The ED offers an opportunity to increase access to MOUD for individuals with OUD. This protocol describes a trial comparing the effects of a standard educational activity to IF on the implementation of ED-initiated buprenorphine with referral for ongoing MOUD. We assess its effectiveness in engaging patients with OUD in formal addiction treatment at 30 days. Vital components of the IF intervention promoting the adoption of ED-initiated buprenorphine will be developed and used. Challenges and solutions to overcoming provider, organization, and perhaps patient barriers will be identified. Targeted interventions and site variations will be identified and addressed. The hybrid design allows for testing the implementation strategy and assessing patient outcomes. This study will provide resources and a roadmap for other EDs. Treatment pathways and algorithms, integration into ED flow, EHR adaptations, and referral strategies and sources will be shared widely.

## Limitations

While geographically diverse, the sites are all large academic centers located in urban environments and external validity in smaller, community hospital EDs in rural and resource limited settings is unknown. However, NIDA CTN trial (0079) is currently conducting parallel work in such settings. In addition, temporal changes are expected to occur over the course of the study due to the high visibility of the opioid epidemic and range of federal funds [39] being available to communities to combat the crisis. These important changes are welcome and may influence the implementation process across sites. Finally, as is standard in clinical settings, our provider population will not remain constant over the conduct of the study due to response, staff turn-over, and residents graduating. Our design allows us to conduct multiple cross-sectional assessments of each site’s ED and community providers over time.

## Conclusion

The ED is rapidly being identified as a “24/7/365” site to combat the opioid crisis by offering access to MOUD treatment [40]. Sustainable, evidence-based practice implementation is a complex and challenging process. The current study has the potential to identify an implementation strategy that can be translated to other EDs thereby increasing the adoption of ED-initiated buprenorphine into practice, thus narrowing the gap between OUD identification and treatment [6].

## Additional files

Additional file 1: Site checklist (DOCX 14 kb)

Additional file 2: Focus group scripted narratives (DOCX 16 kb)

Additional file 3: Example of Organizational Readiness to Change Assessment (ORCA) for ED providers Baseline and IF/Post IF and Readiness and Preparedness Rulers Baseline and Follow up. (DOCX 71 kb)

## Abbreviations

ASAM: American Society of Addiction Medicine
CCTN: Center for the Clinical Trials Network
CEA: Cost-effectiveness analysis
COWS: Clinical Opioid Withdrawal Scale
DATA: Drug Addiction Treatment Act
DEA: Drug Enforcement Administration
DSM-5: Diagnostic and Statistical Manual of Mental Disorders, Fifth Edition
ED: Emergency department
EHR: Electronic health record
EQ-5D: EuroQol
FDA: Food and Drug Administration
GEE: Generalized estimating equation
IF: Implementation facilitation
MOUD: Medications for opioid use disorder
NIDA: National Institute on Drug Abuse
ORCA: Organizational Readiness to Change Assessment
OTP: Opioid treatment program
OUD: Opioid use disorder
PARiHS: Promoting Action on Research Implementation in Health Services
PHQ9: Patient Health Questionnaire
PI: Principal investigator
QALY: Quality-adjusted life years
RA: Research associate
TLFB: Timeline followback
