# Supplementary material for: Implementation facilitation to promote emergency department-initiated buprenorphine for opioid use disorder: protocol for a hybrid type III effectiveness-implementation study (Project ED HEALTH)
Source: Implement Sci. 2019 May 7;14:48. doi: 10.1186/s13012-019-0891-5 (PMC6505286; doi:10.1186/s13012-019-0891-5)
Supplement: Supplementary file 1 — Site checklist (DOCX 14 kb) [file 13012_2019_891_MOESM1_ESM.docx]

| **Additional File 1**  **IF Site Checklist** | | | |
| --- | --- | --- | --- |
| **Completed by Site Champion** | Date | Date | Date |
| **ED Provider Information** | | | |
| No. with an X-waiver |  |  |  |
| No. who have completed buprenorphine training |  |  |  |
| No. who have prescribed buprenorphine |  |  |  |
| No. who have administered buprenorphine |  |  |  |
| Clinical protocol completed (Yes/no) |  |  |  |
| **Referral Information** |  |  |  |
| Agreements with follow up sites made  *(Specify type, OTP, OBP)* |  |  |  |
| **Process Information** |  |  |  |
| Specific IT updates to electronic record  [e.g., clinical decision tools, dot phrases, discharge instructions]  *(Describe changes)* |  |  |  |
| Specify other providers in the ED that assist with the care and referral of patients with OUD such as counselors, social workers, recovery coaches, health advocates, navigators, community health workers? *(Specific each role and #)* |  |  |  |
| Describe education events and learning opportunities pertaining to OUD. For example, Journal club with JAMA 2015 ED buprenorphine article (JAMA 2015 313(16):1636-44), didactic lectures, case studies, on-line tools?  *(Specify audience and venue)* |  |  |  |
| Has a task force related to opioids been established?  If so what disciplines are represented? |  |  |  |
| Is there a CQI process for audit & feedback of ED provider clinical care to monitor the extent to which they prescribe buprenorphine? |  |  |  |
| Is buprenorphine stored in the ED Pyxis? |  |  |  |
| What meetings have occurred between community providers and ED staff to discuss ED-initiated buprenorphine and referral for ongoing MOUD |  |  |  |
|  |  |  |  |
| **Completed by Investigators** |  |  |  |
| Site invited to participate in the Learning Collaborative? |  |  |  |
| Number of individuals provided with academic detailing? |  |  |  |
| How have clinical champions been engaged? (collaborative call etc.) |  |  |  |
