# Supplementary material for: Implementation facilitation to promote emergency department-initiated buprenorphine for opioid use disorder: protocol for a hybrid type III effectiveness-implementation study (Project ED HEALTH)
Source: Implement Sci. 2019 May 7;14:48. doi: 10.1186/s13012-019-0891-5 (PMC6505286; doi:10.1186/s13012-019-0891-5)
Supplement: Supplementary file 2 — Focus group scripted narratives (DOCX 16 kb) [file 13012_2019_891_MOESM2_ESM.docx]

| **Additional File 2: Focus Group Scripted Narratives** |
| --- |
| **ED Providers** |
| 1. **To get started, can you tell me generally about your experiences with treating patients**   **with opioid use disorder or opioid addiction?**   - 1. *To what extent do you think opioid use disorder impact your patients?*   2. *Have you ever prescribed buprenorphine?*   3. *Have you ever referred a patient for buprenorphine or other MOUD(e.g. methadone, naltrexone)?* |
| 1. **Can you tell me, what is your understanding regarding why it makes sense to start**   **buprenorphine in the ED for patients with opioid use disorder and then refer them for**  **ongoing treatment?**   - 1. *What type of evidence comes to mind? (Clinical trials, observational studies, guidelines)*   2. *How relevant do you think this evidence is for your patients?*   *What makes it more/less relevant for your patients?*   - 1. ****What are barriers to initiating buprenorphine in your ED and referring patients for*   *ongoing treatment? What make is easier to implement this practice?*   - 1. ****What type of support do you have to apply this evidence?* |
| 1. **What about your ED directors, administrators and those who pay for the care your**   **patients receive – to what extent do you think they are aware of the evidence supporting**  **ED-initiated buprenorphine with referral for ongoing MOUD for patients with opioid use disorder?**   - 1. *How much do you think your clinical directors and administrators see this as an important issue?*   2. *What do you think impacts how they prioritize this evidence?*   3. *What type of data are collected locally about your patients with opioid use disorder?* |
| 1. **How do you think adopting ED-initiated buprenorphine with referral for ongoing MOUD**   **for patients with opioid use disorder in your ED would impact practice?**   - 1. *What would make it hard to do this? What would make it easier to do this?*   2. *How important do you think it is to do this in your ED?*   3. *What training would you need to do this?*   4. *What systems would you need in place to do this?*   5. *What types of staff changes would you need in your ED to do this?*   6. *What types of performance measures are currently in place?*   7. *What kind of feedback do you get about this to inform your practice?* |
| 1. **What types of resources do you currently have for treating opioid use disorder for**   **patients seen in your ED? And for initiating buprenorphine? Referring patients for**  **ongoing treatment?** |
| **ED Patients with OUD** |
| 1. **To get started, can you tell me generally about your experiences getting treatment at**   **the XX ED?** |
| 1. **Can you tell me, what is your understanding of buprenorphine or “suboxone” as a**   **potential treatment for opioid addiction? What about getting buprenorphine in the ED?**   - 1. What do you think makes this relevant for you or others seen in the ED?   2. What do you think makes this less relevant for you or others seen in the ED? |
| 1. **What about your doctors or other health care providers – what do you think they know**   **about this? How much do they talk to you about your opioid use? and treatment options?**   - 1. *What kinds of conversations to you have about these issues?*   2. *What about buprenorphine specifically?*   3. *What about referral for medication for your addiction?* |
| 1. *****What do you think would be the best way starting someone with an opioid addiction**   **on buprenorphine when they come to the ED – what would it look like?**   - 1. *When would you start the buprenorphine?*   2. *How would you respond if someone prescribed buprenorphine to you?*   *would you want to talk to a doctor or a nurse or counselor?*   - 1. *How many days of medication would you want prescribed?*   2. *What else would be helpful?*   3. *What kind of information would you need?*   4. *What about continuing medication through a program? Where would you go?*   *Would you prefer to go to a methadone program or doctor’s office?* |
| **Community Program Providers** |
| 1. **To get started, can you tell me generally about your understanding of how much there**   **is a need for treatment of opioid use disorder among patients in your community?**   - 1. *To what extent do you think opioid use disorder impact your patients?*   2. *Have you ever prescribed buprenorphine?*   3. *Have you ever been referred patients from your local Emergency Department who have*   *been started on buprenorphine? And what about any patients who had*  *initiated buprenorphine or other MOUD (e.g. methadone, naltrexone)?* |
| 1. **Can you tell me, what is your understanding regarding why it makes sense to**   **start buprenorphine in the ED for patients with opioid use disorder and then refer them**  **for ongoing treatment?**   - 1. *What type of evidence comes to mind? (Clinical trials, observational studies, guidelines)*   2. *How relevant do you think this evidence is for your patients?*   *What makes it more/less relevant for your patients?*   - 1. ****What motivates you to apply this evidence? What makes it hard to apply this evidence?*   2. ****What type of support do you have to apply this evidence?* |
| 1. **What about the staff at your local ED, including ED directors, administrators and those who**   **pay for the care your patients receive – to what extent do you think they are aware of**  **data supporting ED-initiated buprenorphine with referral for ongoing MOUD for patients**  **with opioid use disorder?**   - 1. *How much do you think your local ED’s clinical directors and administrators see*   *treatment of opioid use disorder with buprenorphine as an important issue?*   - 1. *What do you think impacts how they prioritize this evidence?*   2. *What type of data are collected locally about your patients with opioid use disorder?* |
| 1. **What do you think about being referred patients from your local ED for treatment of an**   **opioid use disorder? What about patients who have been started on buprenorphine?**   - 1. *What would make it hard to do this? What would make it easier to do this?*   2. *How important do you think it is to do this (i.e. receive referrals form the ED for patients*   *with opioid use disorder)?*   - 1. *What training would you need to do this?*   2. *What systems would you need in place to do this?*   3. *What types of staff changes would you need in your program to do this?*   4. *What types of performance measures are currently in place? What kind of feedback do*   *you get about this to inform your practice?* |
