# Supplementary material for: Implementation facilitation to promote emergency department-initiated buprenorphine for opioid use disorder: protocol for a hybrid type III effectiveness-implementation study (Project ED HEALTH)
Source: Implement Sci. 2019 May 7;14:48. doi: 10.1186/s13012-019-0891-5 (PMC6505286; doi:10.1186/s13012-019-0891-5)
Supplement: Supplementary file 3 — Example of Organizational Readiness to Change Assessment (ORCA) for ED providers Baseline and IF/Post IF and Readiness and Preparedness Rulers Baseline and Follow up. (DOCX 71 kb) [file 13012_2019_891_MOESM3_ESM.docx]

**Additional File 3: Baseline and Follow-up IF surveys**

**Implementation Facilitation- Baseline**

[Pre IF]

**Organizational Readiness to Change Assessment**

**for ED Providers/Staff (ORCA)**

*We need your help assessing your and your colleagues’ readiness to implement ED-initiated buprenorphine with referral for ongoing medication assisted treatment (MAT) for opioid use disorder in your Emergency Department (ED). By opioid use disorder we mean patients with uncontrolled use (addiction) of illicit (e.g. heroin) or prescription opioids. By MAT, we mean use of medications for the treatment of opioid use disorder. (i.e. buprenorphine, methadone, naltrexone). By treatment engagement, we mean that the patient is receiving treatment for their opioid use disorder with MAT. In the Evidence section below, we ask you to provide your opinions regarding buprenorphine. In the Context section that follows, we ask some questions about features of your ED. Please consider each question carefully and select the answer that best reflects your opinion.*

1. **EVIDENCE ASSESSMENT FOR EACH INTERVENTION**

The following set of questions are about the evidence that ED-initiated buprenorphine with referral for ongoing medication assisted treatment (MAT) to community-based practices/programs improves treatment engagement. For each of the following statements, please rate the strength of your agreement with the statement from 1 (strongly disagree) to 5 (strongly agree).

**IF Evidence Assessment: Buprenorphine FOR OPIOID use disorder**

1. In my opinion, ED-initiated buprenorphine in my ED with referral for ongoing MAT to community-based practices/programs will improve treatment engagement among patients with opioid use disorder.

| Strongly Disagree | Disagree | Neither Agree nor Disagree | Agree | Strongly Agree | Don’t Know | Not Applicable |
| --- | --- | --- | --- | --- | --- | --- |
| 1 | 2 | 3 | 4 | 5 | 98 | 99 |

1. Respected clinical experts in my institution feel that ED-initiated buprenorphine in my ED with referral for ongoing MAT to community-based practices/programs will improve treatment engagement among patients with opioid use disorder.

| Strongly Disagree | Disagree | Neither Agree nor Disagree | Agree | Strongly Agree | Don’t Know | Not Applicable |
| --- | --- | --- | --- | --- | --- | --- |
| 1 | 2 | 3 | 4 | 5 | 98 | 99 |

1. Changes to improve and systematize ED-initiated buprenorphine in my ED with referral for ongoing MAT to community-based practices/programs to promote treatment engagement among patients with opioid use disorder:
   1. are supported by randomized controlled trials (RCTs) or other scientific evidence from my ED

| Strongly Disagree | Disagree | Neither Agree nor Disagree | Agree | Strongly Agree | Don’t Know | Not Applicable |
| --- | --- | --- | --- | --- | --- | --- |
| 1 | 2 | 3 | 4 | 5 | 98 | 99 |

- 1. are supported by randomized controlled trials (RCTs) or other scientific evidence from other health care systems

| Strongly Disagree | Disagree | Neither Agree nor Disagree | Agree | Strongly Agree | Don’t Know | Not Applicable |
| --- | --- | --- | --- | --- | --- | --- |
| 1 | 2 | 3 | 4 | 5 | 98 | 99 |

- 1. should be effective, based on current scientific knowledge

| Strongly Disagree | Disagree | Neither Agree nor Disagree | Agree | Strongly Agree | Don’t Know | Not Applicable |
| --- | --- | --- | --- | --- | --- | --- |
| 1 | 2 | 3 | 4 | 5 | 98 | 99 |

1. ED-initiated buprenorphine in my ED with referral for ongoing MAT to community-based practices/programs to promote treatment engagement among patients with an opioid use disorder:
   1. is supported by clinical experience with my ED’s patients

| Strongly Disagree | Disagree | Neither Agree nor Disagree | Agree | Strongly Agree | Don’t Know | Not Applicable |
| --- | --- | --- | --- | --- | --- | --- |
| 1 | 2 | 3 | 4 | 5 | 98 | 99 |

- 1. is supported by clinical experiences with patients in other health care systems

| Strongly Disagree | Disagree | Neither Agree nor Disagree | Agree | Strongly Agree | Don’t Know | Not Applicable |
| --- | --- | --- | --- | --- | --- | --- |
| 1 | 2 | 3 | 4 | 5 | 98 | 99 |

- 1. conforms to the opinions of clinical experts in this setting

| Strongly Disagree | Disagree | Neither Agree nor Disagree | Agree | Strongly Agree | Don’t Know | Not Applicable |
| --- | --- | --- | --- | --- | --- | --- |
| 1 | 2 | 3 | 4 | 5 | 98 | 99 |

1. ED-initiated buprenorphine in my ED with referral for ongoing MAT to community-based practices/programs will improve health outcomes among patients with an opioid use disorder:
   1. has been well-accepted by my ED’s patients in a pilot study

| Strongly Disagree | Disagree | Neither Agree nor Disagree | Agree | Strongly Agree | Don’t Know | Not Applicable |
| --- | --- | --- | --- | --- | --- | --- |
| 1 | 2 | 3 | 4 | 5 | 98 | 99 |

- 1. is consistent with clinical practices that have been accepted by my ED’s patients

| Strongly Disagree | Disagree | Neither Agree nor Disagree | Agree | Strongly Agree | Don’t Know | Not Applicable |
| --- | --- | --- | --- | --- | --- | --- |
| 1 | 2 | 3 | 4 | 5 | 98 | 99 |

- 1. take into consideration the needs and preferences of my ED’s patients

| Strongly Disagree | Disagree | Neither Agree nor Disagree | Agree | Strongly Agree | Don’t Know | Not Applicable |
| --- | --- | --- | --- | --- | --- | --- |
| 1 | 2 | 3 | 4 | 5 | 98 | 99 |

- 1. appear to have more advantages than disadvantages for my ED’s patients

| Strongly Disagree | Disagree | Neither Agree nor Disagree | Agree | Strongly Agree | Don’t Know | Not Applicable |
| --- | --- | --- | --- | --- | --- | --- |
| 1 | 2 | 3 | 4 | 5 | 98 | 99 |

**II. CONTEXT ASSESSMENT**

The following set of questions is about your experiences in your ED. For each of the following statements, please indicate how frequently you have observed the following sets of behaviors, from 1 (very infrequently) to 5 (very frequently).

1. How frequently have you observed senior leadership/clinical management (e.g. medical director) in your ED:
   1. reward clinical innovation and creativity to improve patient care

| Very Infrequently | Infrequently | Neither Frequently nor Infrequently | Frequently | Very Frequently | Don’t Know | Not Applicable |
| --- | --- | --- | --- | --- | --- | --- |
| 1 | 2 | 3 | 4 | 5 | 98 | 99 |

- 1. solicit opinions of clinical staff regarding decisions about patient care

| Very Infrequently | Infrequently | Neither Frequently nor Infrequently | Frequently | Very Frequently | Don’t Know | Not Applicable |
| --- | --- | --- | --- | --- | --- | --- |
| 1 | 2 | 3 | 4 | 5 | 98 | 99 |

- 1. seek ways to improve patient education and increase patient participation in treatment

| Very Infrequently | Infrequently | Neither Frequently nor Infrequently | Frequently | Very Frequently | Don’t Know | Not Applicable |
| --- | --- | --- | --- | --- | --- | --- |
| 1 | 2 | 3 | 4 | 5 | 98 | 99 |

1. How frequently have you observed staff members in your ED:
   1. have a sense of personal responsibility for improving patient care and outcomes

| Very Infrequently | Infrequently | Neither Frequently nor Infrequently | Frequently | Very Frequently | Don’t Know | Not Applicable |
| --- | --- | --- | --- | --- | --- | --- |
| 1 | 2 | 3 | 4 | 5 | 98 | 99 |

- 1. cooperate to maintain and improve effectiveness of patient care

| Very Infrequently | Infrequently | Neither Frequently nor Infrequently | Frequently | Very Frequently | Don’t Know | Not Applicable |
| --- | --- | --- | --- | --- | --- | --- |
| 1 | 2 | 3 | 4 | 5 | 98 | 99 |

- 1. be willing to innovate and/or experiment to improve clinical procedures

| Very Infrequently | Infrequently | Neither Frequently nor Infrequently | Frequently | Very Frequently | Don’t Know | Not Applicable |
| --- | --- | --- | --- | --- | --- | --- |
| 1 | 2 | 3 | 4 | 5 | 98 | 99 |

- 1. be receptive to change in clinical processes

| Very Infrequently | Infrequently | Neither Frequently nor Infrequently | Frequently | Very Frequently | Don’t Know | Not Applicable |
| --- | --- | --- | --- | --- | --- | --- |
| 1 | 2 | 3 | 4 | 5 | 98 | 99 |

1. How frequently have you observed senior leadership/clinical management (e.g. medical director) in your ED:
   1. provide effective management for continuous improvement of patient care

| Very Infrequently | Infrequently | Neither Frequently nor Infrequently | Frequently | Very Frequently | Don’t Know | Not Applicable |
| --- | --- | --- | --- | --- | --- | --- |
| 1 | 2 | 3 | 4 | 5 | 98 | 99 |

- 1. clearly define areas of responsibility and authority for clinical managers and staff

| Very Infrequently | Infrequently | Neither Frequently nor Infrequently | Frequently | Very Frequently | Don’t Know | Not Applicable |
| --- | --- | --- | --- | --- | --- | --- |
| 1 | 2 | 3 | 4 | 5 | 98 | 99 |

- 1. promote team building to solve clinical care problems

| Very Infrequently | Infrequently | Neither Frequently nor Infrequently | Frequently | Very Frequently | Don’t Know | Not Applicable |
| --- | --- | --- | --- | --- | --- | --- |
| 1 | 2 | 3 | 4 | 5 | 98 | 99 |

- 1. promote communication among clinical services and units

| Very Infrequently | Infrequently | Neither Frequently nor Infrequently | Frequently | Very Frequently | Don’t Know | Not Applicable |
| --- | --- | --- | --- | --- | --- | --- |
| 1 | 2 | 3 | 4 | 5 | 98 | 99 |

1. How frequently have you observed senior leadership/clinical management (e.g. medical director) in your ED:
   1. provide staff with information on your ED’s performance measures and guidelines

| Very Infrequently | Infrequently | Neither Frequently nor Infrequently | Frequently | Very Frequently | Don’t Know | Not Applicable |
| --- | --- | --- | --- | --- | --- | --- |
| 1 | 2 | 3 | 4 | 5 | 98 | 99 |

- 1. establish clear goals for patient care processes and outcomes

| Very Infrequently | Infrequently | Neither Frequently nor Infrequently | Frequently | Very Frequently | Don’t Know | Not Applicable |
| --- | --- | --- | --- | --- | --- | --- |
| 1 | 2 | 3 | 4 | 5 | 98 | 99 |

- 1. provide staff members with feedback/data on effects of clinical decisions

| Very Infrequently | Infrequently | Neither Frequently nor Infrequently | Frequently | Very Frequently | Don’t Know | Not Applicable |
| --- | --- | --- | --- | --- | --- | --- |
| 1 | 2 | 3 | 4 | 5 | 98 | 99 |

- 1. hold staff members accountable for achieving results

| Very Infrequently | Infrequently | Neither Frequently nor Infrequently | Frequently | Very Frequently | Don’t Know | Not Applicable |
| --- | --- | --- | --- | --- | --- | --- |
| 1 | 2 | 3 | 4 | 5 | 98 | 99 |

1. How frequently have you observed opinion leaders in your ED:
   1. express belief that the current practice patterns can be improved

| Very Infrequently | Infrequently | Neither Frequently nor Infrequently | Frequently | Very Frequently | Don’t Know | Not Applicable |
| --- | --- | --- | --- | --- | --- | --- |
| 1 | 2 | 3 | 4 | 5 | 98 | 99 |

- 1. encourage and support changes in practice patterns to improve patient care

| Very Infrequently | Infrequently | Neither Frequently nor Infrequently | Frequently | Very Frequently | Don’t Know | Not Applicable |
| --- | --- | --- | --- | --- | --- | --- |
| 1 | 2 | 3 | 4 | 5 | 98 | 99 |

- 1. demonstrate willingness to try new clinical protocols

| Very Infrequently | Infrequently | Neither Frequently nor Infrequently | Frequently | Very Frequently | Don’t Know | Not Applicable |
| --- | --- | --- | --- | --- | --- | --- |
| 1 | 2 | 3 | 4 | 5 | 98 | 99 |

- 1. work cooperatively with senior leadership/clinical management (e.g. medical director) to make appropriate changes

| Very Infrequently | Infrequently | Neither Frequently nor Infrequently | Frequently | Very Frequently | Don’t Know | Not Applicable |
| --- | --- | --- | --- | --- | --- | --- |
| 1 | 2 | 3 | 4 | 5 | 98 | 99 |

1. In general in your ED, when there is agreement that change needs to happen, how frequently have you or your colleagues:
   1. had the necessary support in terms of budget or financial resources

| Very Infrequently | Infrequently | Neither Frequently nor Infrequently | Frequently | Very Frequently | Don’t Know | Not Applicable |
| --- | --- | --- | --- | --- | --- | --- |
| 1 | 2 | 3 | 4 | 5 | 98 | 99 |

- 1. had the necessary support in terms of training

| Very Infrequently | Infrequently | Neither Frequently nor Infrequently | Frequently | Very Frequently | Don’t Know | Not Applicable |
| --- | --- | --- | --- | --- | --- | --- |
| 1 | 2 | 3 | 4 | 5 | 98 | 99 |

- 1. had the necessary support in terms of facilities

| Very Infrequently | Infrequently | Neither Frequently nor Infrequently | Frequently | Very Frequently | Don’t Know | Not Applicable |
| --- | --- | --- | --- | --- | --- | --- |
| 1 | 2 | 3 | 4 | 5 | 98 | 99 |

- 1. had the necessary support in terms of staffing

| Very Infrequently | Infrequently | Neither Frequently nor Infrequently | Frequently | Very Frequently | Don’t Know | Not Applicable |
| --- | --- | --- | --- | --- | --- | --- |
| 1 | 2 | 3 | 4 | 5 | 98 | 99 |

# Implementation Facilitation (IF)- Baseline

[Pre IF]

**Readiness Ruler – Community Treatment Program/Provider**

**and Staff Survey**

**Instructions**:

The purpose of this study is to learn some basic characteristics of the providers and staff working at your practice/program and models of care for treating opioid use disorder. This survey should take you Papproximately 10 minutes to complete. By clicking the forward arrows at the bottom of this screen, you are agreeing to take part in this study and understand that your responses will be used for research purposes. This study is being administered through the Yale University School of Medicine and has received Institutional Review Board (IRB) approval from Western Institutional Review Board (WIRB), Yale University School of Medicine and each of the participating sites. Your participation is completely voluntary and you do not have to participate if you do not want to; also you may skip questions you do not feel comfortable answering. Your responses will be kept confidential and will only be shared in aggregate and will not impact your job role.

Browsers at work may have different security settings. If you experience any difficulties being directed to the survey or during the survey, try to open the link to the survey in a different browser or using a non-work computer or network. We also recommend that you complete this survey on a desktop computer or laptop. Please contact XXX (Local PI/PD) ([email@xx.edu](mailto:email@xx.edu)) with any questions or concerns.

1. On a scale from 1 to 10, how prepared are you to continue medication assisted treatment (MAT) for patients with an opioid use disorder who have received ED-initiated buprenorphine, where 1 equals “not prepared at all” and 10 equals “totally prepared?” *(knowledge and ability)*

| 1 | 2 | 3 | 4 | 5 | 6 | 7 | 8 | 9 | 10 |
| --- | --- | --- | --- | --- | --- | --- | --- | --- | --- |
| Not prepared at all Unsure Totally Prepared | | | | | | | | | |

1. On a scale from 1 to 10, how ready are you to continue MAT for patients with an opioid use disorder who have received ED-initiated buprenorphine, where 1 equals “not ready at all” and 10 equals “totally ready?” (*willing to provide*)

| 1 | 2 | 3 | 4 | 5 | 6 | 7 | 8 | 9 | 10 |
| --- | --- | --- | --- | --- | --- | --- | --- | --- | --- |
| Not Ready at all Unsure Totally Ready | | | | | | | | | |

**Implementation Facilitation- Follow Up**

[Post Implementation Facilitation(IF)/Post IF Evaluation]

**Organizational Readiness to Change Assessment**

**for ED Providers/Staff (ORCA+)**

*We need your help assessing your and your colleagues’ readiness to implement ED-initiated buprenorphine with referral for ongoing medication assisted treatment (MAT) for opioid use disorder in your Emergency Department (ED). By opioid use disorder we mean patients with uncontrolled use (addiction) of illicit (e.g. heroin) or prescription opioids. By MAT, we mean use of medications for the treatment of opioid use disorder. (i.e. buprenorphine, methadone, naltrexone). By treatment engagement, we mean that the patient is receiving treatment for their opioid use disorder with MAT. In the Evidence section below, we ask you to provide your opinions regarding buprenorphine. In the Context section that follows, we ask some questions about features of your ED. Lastly, in the Facilitation section, we ask you about your opinions regarding the new program to promote ED-initiated buprenorphine in your ED with referral for ongoing MAT to community-based practices/programs.. Please consider each question carefully and select the answer that best reflects your opinion.*

1. **EVIDENCE ASSESSMENT FOR EACH INTERVENTION**

The following set of questions are about the evidence that ED-initiated buprenorphine with referral for ongoing medication assisted treatment (MAT) to community-based practices/programs improves treatment engagement. For each of the following statements, please rate the strength of your agreement with the statement from 1 (strongly disagree) to 5 (strongly agree).

**IF Evidence Assessment: Buprenorphine FOR OPIOID use disorder**

1. In my opinion, ED-initiated buprenorphine in my ED with referral for ongoing MAT to community-based practices/programs will improve treatment engagement among patients with opioid use disorder.

| Strongly Disagree | Disagree | Neither Agree nor Disagree | Agree | Strongly Agree | Don’t Know | Not Applicable |
| --- | --- | --- | --- | --- | --- | --- |
| 1 | 2 | 3 | 4 | 5 | 98 | 99 |

1. Respected clinical experts in my institution feel that ED-initiated buprenorphine in my ED with referral for ongoing MAT to community-based practices/programs will improve treatment engagement among patients with opioid use disorder.

| Strongly Disagree | Disagree | Neither Agree nor Disagree | Agree | Strongly Agree | Don’t Know | Not Applicable |
| --- | --- | --- | --- | --- | --- | --- |
| 1 | 2 | 3 | 4 | 5 | 98 | 99 |

1. Changes to improve and systematize ED-initiated buprenorphine in my ED with referral for ongoing MAT to community-based practices/programs to promote treatment engagement among patients with opioid use disorder:
   1. are supported by randomized controlled trials (RCTs) or other scientific evidence from my ED

| Strongly Disagree | Disagree | Neither Agree nor Disagree | Agree | Strongly Agree | Don’t Know | Not Applicable |
| --- | --- | --- | --- | --- | --- | --- |
| 1 | 2 | 3 | 4 | 5 | 98 | 99 |

- 1. are supported by randomized controlled trials (RCTs) or other scientific evidence from other health care systems

| Strongly Disagree | Disagree | Neither Agree nor Disagree | Agree | Strongly Agree | Don’t Know | Not Applicable |
| --- | --- | --- | --- | --- | --- | --- |
| 1 | 2 | 3 | 4 | 5 | 98 | 99 |

- 1. should be effective, based on current scientific knowledge

| Strongly Disagree | Disagree | Neither Agree nor Disagree | Agree | Strongly Agree | Don’t Know | Not Applicable |
| --- | --- | --- | --- | --- | --- | --- |
| 1 | 2 | 3 | 4 | 5 | 98 | 99 |

1. ED-initiated buprenorphine in my ED with referral for ongoing MAT to community-based practices/programs to promote treatment engagement among patients with an opioid use disorder:
   1. is supported by clinical experience with my ED’s patients

| Strongly Disagree | Disagree | Neither Agree nor Disagree | Agree | Strongly Agree | Don’t Know | Not Applicable |
| --- | --- | --- | --- | --- | --- | --- |
| 1 | 2 | 3 | 4 | 5 | 98 | 99 |

- 1. is supported by clinical experiences with patients in other health care systems

| Strongly Disagree | Disagree | Neither Agree nor Disagree | Agree | Strongly Agree | Don’t Know | Not Applicable |
| --- | --- | --- | --- | --- | --- | --- |
| 1 | 2 | 3 | 4 | 5 | 98 | 99 |

- 1. conforms to the opinions of clinical experts in this setting

| Strongly Disagree | Disagree | Neither Agree nor Disagree | Agree | Strongly Agree | Don’t Know | Not Applicable |
| --- | --- | --- | --- | --- | --- | --- |
| 1 | 2 | 3 | 4 | 5 | 98 | 99 |

1. ED-initiated buprenorphine in my ED with referral for ongoing MAT to community-based practices/programs will improve health outcomes among patients with an opioid use disorder:
   1. has been well-accepted by my ED’s patients in a pilot study

| Strongly Disagree | Disagree | Neither Agree nor Disagree | Agree | Strongly Agree | Don’t Know | Not Applicable |
| --- | --- | --- | --- | --- | --- | --- |
| 1 | 2 | 3 | 4 | 5 | 98 | 99 |

- 1. is consistent with clinical practices that have been accepted by my ED’s patients

| Strongly Disagree | Disagree | Neither Agree nor Disagree | Agree | Strongly Agree | Don’t Know | Not Applicable |
| --- | --- | --- | --- | --- | --- | --- |
| 1 | 2 | 3 | 4 | 5 | 98 | 99 |

- 1. take into consideration the needs and preferences of my ED’s patients

| Strongly Disagree | Disagree | Neither Agree nor Disagree | Agree | Strongly Agree | Don’t Know | Not Applicable |
| --- | --- | --- | --- | --- | --- | --- |
| 1 | 2 | 3 | 4 | 5 | 98 | 99 |

- 1. appear to have more advantages than disadvantages for my ED’s patients

| Strongly Disagree | Disagree | Neither Agree nor Disagree | Agree | Strongly Agree | Don’t Know | Not Applicable |
| --- | --- | --- | --- | --- | --- | --- |
| 1 | 2 | 3 | 4 | 5 | 98 | 99 |

**II. CONTEXT ASSESSMENT**

The following set of questions is about your experiences in your ED. For each of the following statements, please indicate how frequently you have observed the following sets of behaviors, from 1 (very infrequently) to 5 (very frequently).

1. How frequently have you observed senior leadership/clinical management (e.g. medical director) in your ED:
   1. reward clinical innovation and creativity to improve patient care

| Very Infrequently | Infrequently | Neither Frequently nor Infrequently | Frequently | Very Frequently | Don’t Know | Not Applicable |
| --- | --- | --- | --- | --- | --- | --- |
| 1 | 2 | 3 | 4 | 5 | 98 | 99 |

- 1. solicit opinions of clinical staff regarding decisions about patient care

| Very Infrequently | Infrequently | Neither Frequently nor Infrequently | Frequently | Very Frequently | Don’t Know | Not Applicable |
| --- | --- | --- | --- | --- | --- | --- |
| 1 | 2 | 3 | 4 | 5 | 98 | 99 |

- 1. seek ways to improve patient education and increase patient participation in treatment

| Very Infrequently | Infrequently | Neither Frequently nor Infrequently | Frequently | Very Frequently | Don’t Know | Not Applicable |
| --- | --- | --- | --- | --- | --- | --- |
| 1 | 2 | 3 | 4 | 5 | 98 | 99 |

1. How frequently have you observed staff members in your ED:
   1. have a sense of personal responsibility for improving patient care and outcomes

| Very Infrequently | Infrequently | Neither Frequently nor Infrequently | Frequently | Very Frequently | Don’t Know | Not Applicable |
| --- | --- | --- | --- | --- | --- | --- |
| 1 | 2 | 3 | 4 | 5 | 98 | 99 |

- 1. cooperate to maintain and improve effectiveness of patient care

| Very Infrequently | Infrequently | Neither Frequently nor Infrequently | Frequently | Very Frequently | Don’t Know | Not Applicable |
| --- | --- | --- | --- | --- | --- | --- |
| 1 | 2 | 3 | 4 | 5 | 98 | 99 |

- 1. be willing to innovate and/or experiment to improve clinical procedures

| Very Infrequently | Infrequently | Neither Frequently nor Infrequently | Frequently | Very Frequently | Don’t Know | Not Applicable |
| --- | --- | --- | --- | --- | --- | --- |
| 1 | 2 | 3 | 4 | 5 | 98 | 99 |

- 1. be receptive to change in clinical processes

| Very Infrequently | Infrequently | Neither Frequently nor Infrequently | Frequently | Very Frequently | Don’t Know | Not Applicable |
| --- | --- | --- | --- | --- | --- | --- |
| 1 | 2 | 3 | 4 | 5 | 98 | 99 |

1. How frequently have you observed senior leadership/clinical management (e.g. medical director) in your ED:
   1. provide effective management for continuous improvement of patient care

| Very Infrequently | Infrequently | Neither Frequently nor Infrequently | Frequently | Very Frequently | Don’t Know | Not Applicable |
| --- | --- | --- | --- | --- | --- | --- |
| 1 | 2 | 3 | 4 | 5 | 98 | 99 |

- 1. clearly define areas of responsibility and authority for clinical managers and staff

| Very Infrequently | Infrequently | Neither Frequently nor Infrequently | Frequently | Very Frequently | Don’t Know | Not Applicable |
| --- | --- | --- | --- | --- | --- | --- |
| 1 | 2 | 3 | 4 | 5 | 98 | 99 |

- 1. promote team building to solve clinical care problems

| Very Infrequently | Infrequently | Neither Frequently nor Infrequently | Frequently | Very Frequently | Don’t Know | Not Applicable |
| --- | --- | --- | --- | --- | --- | --- |
| 1 | 2 | 3 | 4 | 5 | 98 | 99 |

- 1. promote communication among clinical services and units

| Very Infrequently | Infrequently | Neither Frequently nor Infrequently | Frequently | Very Frequently | Don’t Know | Not Applicable |
| --- | --- | --- | --- | --- | --- | --- |
| 1 | 2 | 3 | 4 | 5 | 98 | 99 |

1. How frequently have you observed senior leadership/clinical management (e.g. medical director) in your ED:
   1. provide staff with information on your ED’s performance measures and guidelines

| Very Infrequently | Infrequently | Neither Frequently nor Infrequently | Frequently | Very Frequently | Don’t Know | Not Applicable |
| --- | --- | --- | --- | --- | --- | --- |
| 1 | 2 | 3 | 4 | 5 | 98 | 99 |

- 1. establish clear goals for patient care processes and outcomes

| Very Infrequently | Infrequently | Neither Frequently nor Infrequently | Frequently | Very Frequently | Don’t Know | Not Applicable |
| --- | --- | --- | --- | --- | --- | --- |
| 1 | 2 | 3 | 4 | 5 | 98 | 99 |

- 1. provide staff members with feedback/data on effects of clinical decisions

| Very Infrequently | Infrequently | Neither Frequently nor Infrequently | Frequently | Very Frequently | Don’t Know | Not Applicable |
| --- | --- | --- | --- | --- | --- | --- |
| 1 | 2 | 3 | 4 | 5 | 98 | 99 |

- 1. hold staff members accountable for achieving results

| Very Infrequently | Infrequently | Neither Frequently nor Infrequently | Frequently | Very Frequently | Don’t Know | Not Applicable |
| --- | --- | --- | --- | --- | --- | --- |
| 1 | 2 | 3 | 4 | 5 | 98 | 99 |

1. How frequently have you observed opinion leaders in your ED:
   1. express belief that the current practice patterns can be improved

| Very Infrequently | Infrequently | Neither Frequently nor Infrequently | Frequently | Very Frequently | Don’t Know | Not Applicable |
| --- | --- | --- | --- | --- | --- | --- |
| 1 | 2 | 3 | 4 | 5 | 98 | 99 |

- 1. encourage and support changes in practice patterns to improve patient care

| Very Infrequently | Infrequently | Neither Frequently nor Infrequently | Frequently | Very Frequently | Don’t Know | Not Applicable |
| --- | --- | --- | --- | --- | --- | --- |
| 1 | 2 | 3 | 4 | 5 | 98 | 99 |

- 1. demonstrate willingness to try new clinical protocols

| Very Infrequently | Infrequently | Neither Frequently nor Infrequently | Frequently | Very Frequently | Don’t Know | Not Applicable |
| --- | --- | --- | --- | --- | --- | --- |
| 1 | 2 | 3 | 4 | 5 | 98 | 99 |

- 1. work cooperatively with senior leadership/clinical management (e.g. medical director) to make appropriate changes

| Very Infrequently | Infrequently | Neither Frequently nor Infrequently | Frequently | Very Frequently | Don’t Know | Not Applicable |
| --- | --- | --- | --- | --- | --- | --- |
| 1 | 2 | 3 | 4 | 5 | 98 | 99 |

1. In general in your ED, when there is agreement that change needs to happen, how frequently have you or your colleagues:
   1. had the necessary support in terms of budget or financial resources

| Very Infrequently | Infrequently | Neither Frequently nor Infrequently | Frequently | Very Frequently | Don’t Know | Not Applicable |
| --- | --- | --- | --- | --- | --- | --- |
| 1 | 2 | 3 | 4 | 5 | 98 | 99 |

- 1. had the necessary support in terms of training

| Very Infrequently | Infrequently | Neither Frequently nor Infrequently | Frequently | Very Frequently | Don’t Know | Not Applicable |
| --- | --- | --- | --- | --- | --- | --- |
| 1 | 2 | 3 | 4 | 5 | 98 | 99 |

- 1. had the necessary support in terms of facilities

| Very Infrequently | Infrequently | Neither Frequently nor Infrequently | Frequently | Very Frequently | Don’t Know | Not Applicable |
| --- | --- | --- | --- | --- | --- | --- |
| 1 | 2 | 3 | 4 | 5 | 98 | 99 |

- 1. had the necessary support in terms of staffing

| Very Infrequently | Infrequently | Neither Frequently nor Infrequently | Frequently | Very Frequently | Don’t Know | Not Applicable |
| --- | --- | --- | --- | --- | --- | --- |
| 1 | 2 | 3 | 4 | 5 | 98 | 99 |

**III. FACILITATION ASSESSMENT**

The following set of questions relates to a recent project to promote ED-initiated buprenorphine in your ED with referral for ongoing MAT to community-based practices/programs, to promote treatment engagement among patients with opioid use disorder. This is referred to as “intervention” below. For each of the following statements, please rate the strength of your agreement with the statement from 1 (strongly disagree) to 5 (strongly agree):

1. For this project, senior leadership/clinical management (e.g. medical director) have:
   1. proposed a project that is appropriate and feasible

| Strongly Disagree | Disagree | Neither Agree nor Disagree | Agree | Strongly Agree | Don’t Know | Not Applicable |
| --- | --- | --- | --- | --- | --- | --- |
| 1 | 2 | 3 | 4 | 5 | 98 | 99 |

- 1. provided clear goals for improvement in patient care

| Strongly Disagree | Disagree | Neither Agree nor Disagree | Agree | Strongly Agree | Don’t Know | Not Applicable |
| --- | --- | --- | --- | --- | --- | --- |
| 1 | 2 | 3 | 4 | 5 | 98 | 99 |

- 1. established a project schedule and deliverables

| Strongly Disagree | Disagree | Neither Agree nor Disagree | Agree | Strongly Agree | Don’t Know | Not Applicable |
| --- | --- | --- | --- | --- | --- | --- |
| 1 | 2 | 3 | 4 | 5 | 98 | 99 |

- 1. designated a clinical champion for the project

| Strongly Disagree | Disagree | Neither Agree nor Disagree | Agree | Strongly Agree | Don’t Know | Not Applicable |
| --- | --- | --- | --- | --- | --- | --- |
| 1 | 2 | 3 | 4 | 5 | 98 | 99 |

1. The project clinical champion:
   1. accepts responsibility for the success of this project

| Strongly Disagree | Disagree | Neither Agree nor Disagree | Agree | Strongly Agree | Don’t Know | Not Applicable |
| --- | --- | --- | --- | --- | --- | --- |
| 1 | 2 | 3 | 4 | 5 | 98 | 99 |

- 1. has the authority to carry out the implementation

| Strongly Disagree | Disagree | Neither Agree nor Disagree | Agree | Strongly Agree | Don’t Know | Not Applicable |
| --- | --- | --- | --- | --- | --- | --- |
| 1 | 2 | 3 | 4 | 5 | 98 | 99 |

- 1. is considered a clinical opinion leader

| Strongly Disagree | Disagree | Neither Agree nor Disagree | Agree | Strongly Agree | Don’t Know | Not Applicable |
| --- | --- | --- | --- | --- | --- | --- |
| 1 | 2 | 3 | 4 | 5 | 98 | 99 |

- 1. works well with the intervention team and providers (i.e. providers prescribing buprenorphine and team implementing referrals for ongoing MAT in your ED)

| Strongly Disagree | Disagree | Neither Agree nor Disagree | Agree | Strongly Agree | Don’t Know | Not Applicable |
| --- | --- | --- | --- | --- | --- | --- |
| 1 | 2 | 3 | 4 | 5 | 98 | 99 |

1. Senior leadership/clinical management/staff opinion leaders:
   1. agree on the goals for this intervention

| Strongly Disagree | Disagree | Neither Agree nor Disagree | Agree | Strongly Agree | Don’t Know | Not Applicable |
| --- | --- | --- | --- | --- | --- | --- |
| 1 | 2 | 3 | 4 | 5 | 98 | 99 |

- 1. will be informed and involved in the intervention

| Strongly Disagree | Disagree | Neither Agree nor Disagree | Agree | Strongly Agree | Don’t Know | Not Applicable |
| --- | --- | --- | --- | --- | --- | --- |
| 1 | 2 | 3 | 4 | 5 | 98 | 99 |

- 1. agree on adequate resources to accomplish the intervention

| Strongly Disagree | Disagree | Neither Agree nor Disagree | Agree | Strongly Agree | Don’t Know | Not Applicable |
| --- | --- | --- | --- | --- | --- | --- |
| 1 | 2 | 3 | 4 | 5 | 98 | 99 |

- 1. set a high priority on the success of the intervention

| Strongly Disagree | Disagree | Neither Agree nor Disagree | Agree | Strongly Agree | Don’t Know | Not Applicable |
| --- | --- | --- | --- | --- | --- | --- |
| 1 | 2 | 3 | 4 | 5 | 98 | 99 |

1. The implementation team members:
   1. share responsibility for the success of this project

| Strongly Disagree | Disagree | Neither Agree nor Disagree | Agree | Strongly Agree | Don’t Know | Not Applicable |
| --- | --- | --- | --- | --- | --- | --- |
| 1 | 2 | 3 | 4 | 5 | 98 | 99 |

- 1. have clearly defined roles and responsibilities

| Strongly Disagree | Disagree | Neither Agree nor Disagree | Agree | Strongly Agree | Don’t Know | Not Applicable |
| --- | --- | --- | --- | --- | --- | --- |
| 1 | 2 | 3 | 4 | 5 | 98 | 99 |

- 1. have release time or can accomplish intervention tasks within their regular work load

| Strongly Disagree | Disagree | Neither Agree nor Disagree | Agree | Strongly Agree | Don’t Know | Not Applicable |
| --- | --- | --- | --- | --- | --- | --- |
| 1 | 2 | 3 | 4 | 5 | 98 | 99 |

- 1. have staff support and other resources required for the project

| Strongly Disagree | Disagree | Neither Agree nor Disagree | Agree | Strongly Agree | Don’t Know | Not Applicable |
| --- | --- | --- | --- | --- | --- | --- |
| 1 | 2 | 3 | 4 | 5 | 98 | 99 |

1. The implementation plan for this intervention:
   1. identifies specific roles and responsibilities

| Strongly Disagree | Disagree | Neither Agree nor Disagree | Agree | Strongly Agree | Don’t Know | Not Applicable |
| --- | --- | --- | --- | --- | --- | --- |
| 1 | 2 | 3 | 4 | 5 | 98 | 99 |

- 1. clearly describes tasks and timelines

| Strongly Disagree | Disagree | Neither Agree nor Disagree | Agree | Strongly Agree | Don’t Know | Not Applicable |
| --- | --- | --- | --- | --- | --- | --- |
| 1 | 2 | 3 | 4 | 5 | 98 | 99 |

- 1. includes appropriate provider/patient education

| Strongly Disagree | Disagree | Neither Agree nor Disagree | Agree | Strongly Agree | Don’t Know | Not Applicable |
| --- | --- | --- | --- | --- | --- | --- |
| 1 | 2 | 3 | 4 | 5 | 98 | 99 |

- 1. acknowledges staff input and opinions

| Strongly Disagree | Disagree | Neither Agree nor Disagree | Agree | Strongly Agree | Don’t Know | Not Applicable |
| --- | --- | --- | --- | --- | --- | --- |
| 1 | 2 | 3 | 4 | 5 | 98 | 99 |

1. Communication will be maintained through:
   1. regular project meetings with the project champion and team members

| Strongly Disagree | Disagree | Neither Agree nor Disagree | Agree | Strongly Agree | Don’t Know | Not Applicable |
| --- | --- | --- | --- | --- | --- | --- |
| 1 | 2 | 3 | 4 | 5 | 98 | 99 |

- 1. involvement of quality management staff in project planning and implementation

| Strongly Disagree | Disagree | Neither Agree nor Disagree | Agree | Strongly Agree | Don’t Know | Not Applicable |
| --- | --- | --- | --- | --- | --- | --- |
| 1 | 2 | 3 | 4 | 5 | 98 | 99 |

- 1. regular feedback to clinical management on progress of project activities and resource needs

| Strongly Disagree | Disagree | Neither Agree nor Disagree | Agree | Strongly Agree | Don’t Know | Not Applicable |
| --- | --- | --- | --- | --- | --- | --- |
| 1 | 2 | 3 | 4 | 5 | 98 | 99 |

- 1. regular feedback to clinicians on effects of practice changes on patient care/outcomes

| Strongly Disagree | Disagree | Neither Agree nor Disagree | Agree | Strongly Agree | Don’t Know | Not Applicable |
| --- | --- | --- | --- | --- | --- | --- |
| 1 | 2 | 3 | 4 | 5 | 98 | 99 |

1. Progress of the project will be measured by:
   1. collecting feedback from patients regarding proposed/implemented changes

| Strongly Disagree | Disagree | Neither Agree nor Disagree | Agree | Strongly Agree | Don’t Know | Not Applicable |
| --- | --- | --- | --- | --- | --- | --- |
| 1 | 2 | 3 | 4 | 5 | 98 | 99 |

- 1. collecting feedback from staff regarding proposed/implemented changes

| Strongly Disagree | Disagree | Neither Agree nor Disagree | Agree | Strongly Agree | Don’t Know | Not Applicable |
| --- | --- | --- | --- | --- | --- | --- |
| 1 | 2 | 3 | 4 | 5 | 98 | 99 |

- 1. developing and distributing regular performance measures to clinical staff

| Strongly Disagree | Disagree | Neither Agree nor Disagree | Agree | Strongly Agree | Don’t Know | Not Applicable |
| --- | --- | --- | --- | --- | --- | --- |
| 1 | 2 | 3 | 4 | 5 | 98 | 99 |

- 1. providing a forum for presentation/discussion of results and implications for continued improvements

| Strongly Disagree | Disagree | Neither Agree nor Disagree | Agree | Strongly Agree | Don’t Know | Not Applicable |
| --- | --- | --- | --- | --- | --- | --- |
| 1 | 2 | 3 | 4 | 5 | 98 | 99 |

1. The following are available to make the select plan work:
   1. staff incentives

| Strongly Disagree | Disagree | Neither Agree nor Disagree | Agree | Strongly Agree | Don’t Know | Not Applicable |
| --- | --- | --- | --- | --- | --- | --- |
| 1 | 2 | 3 | 4 | 5 | 98 | 99 |

- 1. equipment and materials

| Strongly Disagree | Disagree | Neither Agree nor Disagree | Agree | Strongly Agree | Don’t Know | Not Applicable |
| --- | --- | --- | --- | --- | --- | --- |
| 1 | 2 | 3 | 4 | 5 | 98 | 99 |

- 1. patient awareness/need

| Strongly Disagree | Disagree | Neither Agree nor Disagree | Agree | Strongly Agree | Don’t Know | Not Applicable |
| --- | --- | --- | --- | --- | --- | --- |
| 1 | 2 | 3 | 4 | 5 | 98 | 99 |

- 1. provider buy-in

| Strongly Disagree | Disagree | Neither Agree nor Disagree | Agree | Strongly Agree | Don’t Know | Not Applicable |
| --- | --- | --- | --- | --- | --- | --- |
| 1 | 2 | 3 | 4 | 5 | 98 | 99 |

- 1. intervention team (i.e. providers prescribing buprenorphine and team implementing referrals for ongoing MAT in your ED)

| Strongly Disagree | Disagree | Neither Agree nor Disagree | Agree | Strongly Agree | Don’t Know | Not Applicable |
| --- | --- | --- | --- | --- | --- | --- |
| 1 | 2 | 3 | 4 | 5 | 98 | 99 |

- 1. evaluation protocol

| Strongly Disagree | Disagree | Neither Agree nor Disagree | Agree | Strongly Agree | Don’t Know | Not Applicable |
| --- | --- | --- | --- | --- | --- | --- |
| 1 | 2 | 3 | 4 | 5 | 98 | 99 |

1. Plans for evaluation and improvement of this intervention include:
   1. periodic outcome measurement

| Strongly Disagree | Disagree | Neither Agree nor Disagree | Agree | Strongly Agree | Don’t Know | Not Applicable |
| --- | --- | --- | --- | --- | --- | --- |
| 1 | 2 | 3 | 4 | 5 | 98 | 99 |

- 1. staff participation/satisfaction survey

| Strongly Disagree | Disagree | Neither Agree nor Disagree | Agree | Strongly Agree | Don’t Know | Not Applicable |
| --- | --- | --- | --- | --- | --- | --- |
| 1 | 2 | 3 | 4 | 5 | 98 | 99 |

- 1. patient satisfaction survey

| Strongly Disagree | Disagree | Neither Agree nor Disagree | Agree | Strongly Agree | Don’t Know | Not Applicable |
| --- | --- | --- | --- | --- | --- | --- |
| 1 | 2 | 3 | 4 | 5 | 98 | 99 |

- 1. dissemination plan for performance measures

| Strongly Disagree | Disagree | Neither Agree nor Disagree | Agree | Strongly Agree | Don’t Know | Not Applicable |
| --- | --- | --- | --- | --- | --- | --- |
| 1 | 2 | 3 | 4 | 5 | 98 | 99 |

- 1. review of results by clinical leadership

| Strongly Disagree | Disagree | Neither Agree nor Disagree | Agree | Strongly Agree | Don’t Know | Not Applicable |
| --- | --- | --- | --- | --- | --- | --- |
| 1 | 2 | 3 | 4 | 5 | 98 | 99 |

# Implementation Facilitation (IF)- Follow up

[Post IF/ Post IF Evaluation Period]

**Readiness Ruler – Community Treatment Program/Provider**

**and Staff Survey**

**Instructions**:

The purpose of this study is to learn some basic characteristics of the providers and staff working at your practice/program and models of care for treating opioid use disorder. This survey should take you approximately 10 minutes to complete. By clicking the forward arrows at the bottom of this screen, you are agreeing to take part in this study and understand that your responses will be used for research purposes. This study is being administered through the Yale University School of Medicine and has received Institutional Review Board (IRB) approval from Western Institutional Review Board (WIRB), Yale University School of Medicine and each of the participating sites. Your participation is completely voluntary and you do not have to participate if you do not want to; also you may skip questions you do not feel comfortable answering. Your responses will be kept confidential and will only be shared in aggregate and will not impact your job role.

Browsers at work may have different security settings. If you experience any difficulties being directed to the survey or during the survey, try to open the link to the survey in a different browser or using a non-work computer or network. We also recommend that you complete this survey on a desktop computer or laptop. Please contact XXX (Local PI/PD) ([email@xx.edu](mailto:email@xx.edu)) with any questions or concerns.

1. On a scale from 1 to 10, how prepared are you to continue medication assisted treatment (MAT) for patients with an opioid use disorder who have received ED-initiated buprenorphine, where 1 equals “not prepared at all” and 10 equals “totally prepared?” *(knowledge and ability)*

| 1 | 2 | 3 | 4 | 5 | 6 | 7 | 8 | 9 | 10 |
| --- | --- | --- | --- | --- | --- | --- | --- | --- | --- |
| Not prepared at all Unsure Totally Prepared | | | | | | | | | |

1. On a scale from 1 to 10, how ready are you to continue MAT for patients with an opioid use disorder who have received ED-initiated buprenorphine, where 1 equals “not ready at all” and 10 equals “totally ready?” (*willing to provide*)

| 1 | 2 | 3 | 4 | 5 | 6 | 7 | 8 | 9 | 10 |
| --- | --- | --- | --- | --- | --- | --- | --- | --- | --- |
| Not Ready at all Unsure Totally Ready | | | | | | | | | |
